# Airway tapering: an objective image biomarker for bronchiectasis

**DOI:** 10.1007/s00330-019-06606-w

**Published:** 2020-02-05

**Authors:** Wieying Kuo, Adria Perez-Rovira, Harm Tiddens, Marleen de Bruijne, on behalf of the Normal Chest CT study group, Lauren Akesson, Silvia Bertolo, Alan S. Brody, Kris de Boeck, Pim A. de Jong, Robert J. Fleck, Francesco Fraioli, Pilar Garcia-Peña, Silvia Gartner, Edward Y. Lee, Anders Lindblad, Michael McCartin, Christian P. Mol, Giovanni Morana, Arlette E. Odink, Matteo Paoletti, Stephen M. Stick, Els van der Wiel, Francois Vermeulen

**Affiliations:** 1 Department of Paediatric Pulmonology and Allergology, Erasmus MC – Sophia Children’s Hospital, Rotterdam, The Netherlands; 2 Department of Radiology and Nuclear Medicine, Erasmus MC, Rotterdam, The Netherlands; 3 Biomedical Imaging Group Rotterdam, Department of Radiology and Nuclear Medicine, Erasmus MC, room NA-2611, PO Box 2040, 3000 CA Rotterdam, The Netherlands; 4 Department of Computer Science, University of Copenhagen, Copenhagen, Denmark

**Keywords:** Bronchiectasis, Bronchi, Image interpretation computer-assisted, Imaging three-dimensional, Multidetector computed tomography

## Abstract

**Purpose:**

To estimate airway tapering in control subjects and to assess the usability of tapering as a bronchiectasis biomarker in paediatric populations.

**Methods:**

Airway tapering values were semi-automatically quantified in 156 children with control CTs collected in the Normal Chest CT Study Group. Airway tapering as a biomarker for bronchiectasis was assessed on spirometer-guided inspiratory CTs from 12 patients with bronchiectasis and 12 age- and sex-matched controls. Semi-automatic image analysis software was used to quantify intra-branch tapering (reduction in airway diameter along the branch), inter-branch tapering (reduction in airway diameter before and after bifurcation) and airway-artery ratios on chest CTs. Biomarkers were further stratified in small, medium and large airways based on three equal groups of the accompanying vessel size.

**Results:**

Control subjects showed intra-branch tapering of 1% and inter-branch tapering of 24–39%. Subjects with bronchiectasis showed significantly reduced intra-branch of 0.8% and inter-branch tapering of 19–32% and increased airway–artery ratios compared with controls (*p* < 0.01). Tapering measurements were significantly different between diseased and controls across all airway sizes. Difference in airway–artery ratio was only significant in small airways.

**Conclusion:**

Paediatric normal values for airway tapering were established in control subjects. Tapering showed to be a promising biomarker for bronchiectasis as subjects with bronchiectasis show significantly less airway tapering across all airway sizes compared with controls. Detecting less tapering in larger airways could potentially lead to earlier diagnosis of bronchiectasis. Additionally, compared with the conventional airway–artery ratio, this novel biomarker has the advantage that it does not require pairing with pulmonary arteries.

**Key Points:**

• *Tapering is a promising objective image biomarker for bronchiectasis that can be extracted semi-automatically and has good correlation with validated visual scoring methods.*

• *Less airway tapering was observed in patients with bronchiectasis and can be observed sensitively throughout the bronchial tree, even in the more central airways.*

• *Tapering values seemed to be less influenced by variety in scanning protocols and lung volume making it a more robust biomarker for bronchiectasis detection.*

**Electronic supplementary material:**

The online version of this article (10.1007/s00330-019-06606-w) contains supplementary material, which is available to authorized users.

## Introduction

Airway tapering is the reduction in diameter from the central to the peripheral airways. In healthy subjects, the diameter of an airway branch is similar to that of the accompanying artery [1] and there is subtle but progressive diameter reduction along each branch named intra-branch tapering [2]. In addition, the diameter of the airway branch is reduced compared with the airway branch before bifurcation [3] also referred to as inter-branch tapering.

Bronchiectasis is a structural change that can occur in diseased airways of patients with cystic fibrosis (CF) [4, 5], chronic obstructive pulmonary disease (COPD) [4], common variable immunodeficiency (CVID) [6] and tuberculosis [2]. Bronchiectasis is defined as an irreversible dilation of the airways accompanied with lack of tapering [2]. Chest computed tomography (CT) is currently the most sensitive imaging tool to detect bronchiectasis [7, 8] and is a sensitive outcome measure in CF for both clinical and research purposes [9–11]. There are three ways to diagnose bronchiectasis on CT. Firstly, the diameter of the airway exceeds that of the accompanying artery, also quantified by computing the airway–artery ratio (AA ratio) [12–16]. Secondly, visibility of airways within 1 cm of the costal pleura or the mediastinal pleura indicates bronchiectasis. Lastly, lack of tapering, defined as an unchanged airway diameter for 2 cm after branching [2], is a recognised criterion for diagnosing bronchiectasis [17].

Tapering has been included in various semi-quantitative visual scoring systems for bronchiectasis. Nevertheless, only a few scoring systems and studies have included a tapering quantification to assess bronchiectasis in the paediatric population [14, 18].

**Fig. 1:**
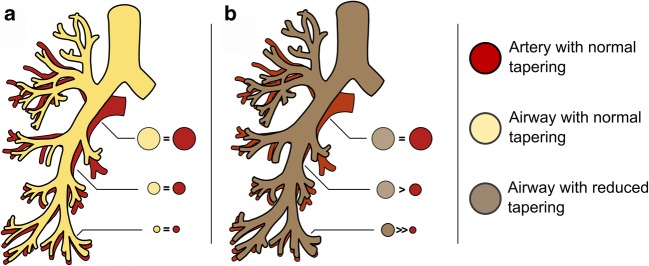
**a** Bronchial tree with tapering similar to its accompanying artery, showing approximately constant airway–artery ratio. **b** Bronchial tree with reduced airway tapering uniformly distributed across the entire bronchial tree, showing an increased airway–artery ratio more pronounced in the more peripheral airways

In this study, we used newly developed state-of-the-art automated image analysis software to (1) estimate control values for intra-branch and inter-branch tapering in peadiatric patients with a normal chest CT; and (2) assess differences in airway tapering between control patients and patients with bronchiectasis, comparing the diagnostic value of airway tapering and AA ratios in children with early to moderate lung disease. We hypothesise that lack of tapering can be quantified using automatic analysis in order to detect bronchiectasis in early to moderate lung disease. Lack of tapering could potentially be detected in the larger airways when a change in AA ratio is not yet prominent. An abnormally dilated peripheral airway might coincide with reduced tapering solely present in the dilated airway branch, in the airway branches immediately before the dilated branch or all the way up to the central airways (Fig. 1).

## Materials and methods

This study was approved by institutional review board (MEC-2014-254 and MEC-2011-494). Written informed consent was waived for all subjects due to the retrospective nature of this study.

### Study population

In this study, a total of 180 CTs from three datasets were used.

Airway tapering values were obtained in a subset of three participating centres from the Normal Chest CT Study Group. This dataset was developed to establish chest CT reference values in children and was reported in a previous publication [19]. In summary, retrospectively collected CTs were made for various clinical indications of paediatric subjects aged 0 to 18 years old. CTs considered to be normal by the reporting radiologist from the initial ten international centres were included after a reassessment by an independent radiologist (C.Y.). All CTs were deidentified and anonymised. In this study, 156 CTs from three centres were included and further referred to as the NormalCT dataset. CTs were acquired during free breathing for non-cooperative young children or a voluntary breath-hold in older subjects. Forty-two of the 156 subjects were scanned with contrast.

The Matched-Bronchiectasis and Matched-Controls datasets were used to assess differences in airway tapering between patients with bronchiectasis and control subjects. The Matched-Bronchiectasis and Matched-Controls datasets have been initially used to study changes in AA ratios and were reported separately [1, 20, 21]. In summary, the Matched-Bronchiectasis consisted of spirometer-guided inspiratory chest CTs of 12 patients between 8 and 16 years old (11 CF and 1 non-CF bronchiectatic patient with CVID; 7 females) with reported signs of bronchiectasis. Matched-Controls consisted of 12 spirometer-guided inspiratory scans of age- and sex-matched controls. The control patients were referred to the Erasmus MC-Sophia Children’s Hospital for a chest CT for several clinical indications and their CT was assessed as normal by the Erasmus MC radiologists and an independent radiologist (C.Y.). Both groups were treated at the Erasmus MC-Sophia Children’s Hospital (electronic supplementary material (ESM) for scanning details).

Visual scores were obtained for all CTs from the matched datasets using the CF-CT scoring module as reported previously [1, 14]. In short, the five lung lobes and lingula were evaluated for severity and extent of bronchiectasis, airway wall thickening and mucous plugging on inspiratory CT. Only bronchiectasis sub-score is reported in the current study.

### Automatic quantification of airways and arteries

In-house developed software (written in C++ and MATLAB) was used to measure AA ratio and airway tapering for all extracted airways. This previously validated method [21] automatically extracts the bronchial tree and arteries using a classifier approach [22]. It obtains diameter measurements every 0.5 mm via a graph-based method [23], and pairs airway and artery branches according to their proximity and similarity in size and orientation. Because of the large variations in inspiration level and scanning protocols in the NormalCT dataset, the automatic extraction of initial airway centrelines was replaced by manually drawn airway centrelines (detailed in ESM). Airway centrelines in the NormalCT dataset were drawn manually as an initialisation after which the segmentation was done automatically. Therefore, the segmentation was fully automatic but the extraction was not. Variations in lung size due to differences in subject size, sex and inspiration level were adjusted for. All measurements were isotropically rescaled, using the cubic root of the lung volume measured on CT, to correspond to 4 L, which is the approximate measured mean lung volume of all subjects.

For each airway artery pair, the following measurements were obtained for the inner and outer airway diameters separately:

#### Airway–artery ratio

Obtained as the ratio between the airway diameter and its accompanying artery diameter. AA ratio was determined along the branch where the outer airway and artery diameters were most similar in terms of size, orientation and position.

#### Intra-branch tapering

**Fig. 2:**
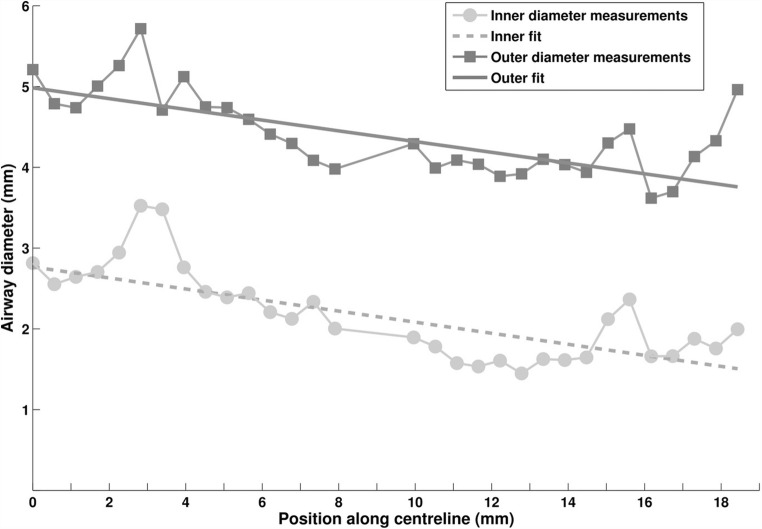
Diameter measurements along an airway branch in a control subject showing progressive reduction of diameter due to intra-branch tapering. Left (0) corresponds to the proximal end just after bifurcation and right (19) to the distal end just before the next bifurcation

A line (*y = mx + n*, where *m* is the slope of the line and *n* the *y*-intercept) was fitted to the diameters measured along the airway branch, using the bi-square weights method (Fig. 2). Intra-branch tapering was obtained as *intraBT =*$\frac{-m}{n}\times 100$, demonstrating the percentage reduction in airway diameter per millimetre along the centreline. Intra-branch tapering (intraBT) of 2 means that the airway diameter is reduced by 2% per millimetre.

#### Inter-branch tapering

Inter-branch tapering was obtained as *interBT =*$\frac{d_p-d}{d_p}\times 100$, where *d* is the average diameter of the analysed airway branch and *d*_*p*_ is the average diameter of the parent airway (the airway branch one generation previously). Inter-branch tapering (interBT) of 20 would indicate that the airway branch is 20% smaller than that before bifurcation.

Tapering was measured for all airways, but for ease of comparison with the AA ratios, only measurements of airways paired with an artery are reported, unless stated otherwise.

### Image biomarkers for bronchiectasis quantification

Per-subject median AA ratio, interBT and intraBT of inner and outer airway were compared between Matched-Bronchiectasis and Matched-Control datasets to assess their value as biomarkers for bronchiectasis.

Previous studies showed that diseased subjects showed more visible peripheral airways on CT compared with control subjects [1, 24]. Since peripheral airways in diseased subjects are larger and may have more clearly defined walls than in controls, peripheral airways that would be too small to be observed if healthy become large enough to be visible on CT. This leads to a selection bias where more peripheral airways are visible in patients with airway disease compared with controls, influencing biomarker differences between controls and diseased. Therefore, differences between groups were also analysed by airway size. All airways from the Matched-Controls dataset were divided into three equally sized groups according to the diameter of their accompanying artery. Airways with an accompanying artery smaller than 3.08 mm, between 3.08 and 4.23 mm and greater than 4.23 mm were considered airways with small, medium and large arteries respectively. These thresholds were also applied to the Matched-Bronchiectasis and NormalCT datasets. Airway generation was acquired automatically for each measurement, but was not used for grouping due to the inconsistency of the airway sizes of the upper segmental (starting at generation 3 to 4) and the lower segmental bronchi (starting at generation 4 to 7) [1].

### Stability of biomarkers under varying scanning conditions

Extracted biomarkers were investigated to variations in scanning conditions. Biomarkers extracted from the single-centre spirometry-controlled inspiration CTs from the Matched-Control dataset were compared with the biomarkers obtained in the CTs from the multicentre NormalCT group, which are of CTs with a large variation in inspiration level and scanning protocols.

### Statistical analysis

In order to exclude outliers and segmentation errors, we performed robust statistics using median and inter-quartile ranges (IQRs). The median was used to summarise all airway–artery measurements per subject and the median and IQR were reported for each population. Per subject median values of each airway size group were compared between all datasets using the Mann–Whitney *U* test. Spearman correlation coefficient was performed to assess correlations. *P* values < 0.05 were considered significant.

## Results

Demographics of all subjects in this study are presented in Table 1. A total of 22,275 airway branches were semi-automatically and 153,238 artery branches were automatically extracted. Of these, 13,987 airway–artery pairs were detected and their AA ratio and tapering measurements were measured automatically for the purpose of this study.

**Table 1:** Demographics and visual scores of the three datasets

	Matched-Bronchiectasis	Matched-Controls	NormalCT
Lung volume during CT acquisition	Spirometer-guided inspiratory CT	Spirometer-guided inspiratory CT	Free breathing and voluntary breath hold
*n*	12	12	156
Age at CT (years old)	10.6 [9.7, 11.7]	13.9 [8.7, 15.0]	10.7 [5.5, 15.0]
Sex	7 males; 5 females	7 males; 5 females	101 males; 54 females
Height (cm)	143.7 [137.4, 146.2]	149 [136.6, 170.9]	Not available
Weight (kg)	34.3 [29.5, 40.8]	40.1 [28.6, 65.8]	Not available
BMI	17.2 [15.6, 18.5]	18.1 [15.9, 20.2]	Not available
CFCT BE (%)	7.5 [1.4, 13.3]	0.0 [0.0, 0.7]*	Not available

The demographics and visual scores are expressed as median [lower quantile, upper quartile]

*CFCT BE* stands for the visual sub-scoring of bronchiectasis

*Statistically significant differences with Matched-Bronchiectasis (*p* value < 0.05)

### Control values

In the NormalCT dataset, median intraBT of the inner and intraBT of the outer airways were 1.16% and 0.94%, respectively, expressed as a percentage reduction in airway diameter per millimetre. Median interBT of the inner and interBT of the outer airways were 38.54% and 23.75%, respectively, expressed as the percentage diameter reduction for each airway branching.

Stratified by artery size for small, medium and large, intraBT of the inner airway were 1.33%, 1.33% and 0.95%; intraBT of the outer airway were 0.94%, 1.00% and 0.93%; interBT of the inner airway were 42.02%, 39.35% and 34.20%; and interBT of the outer airway were 25.39%, 25.08% and 22.08%, respectively (ESM e-Tables 2–4 for details).

### Image biomarkers for bronchiectasis quantification

Matched-Bronchiectasis did not show a significant difference in inner AA ratio compared with Matched-Controls (*p* < 0.470). Outer AA ratio was significantly increased (*p* < 0.022) in patients with bronchiectasis compared with that in controls.

All inter-branch and intra-branch tapering values were significantly lower (*p* < 0.003) in patients with bronchiectasis compared with those in the matched controls.

**Fig. 3:**
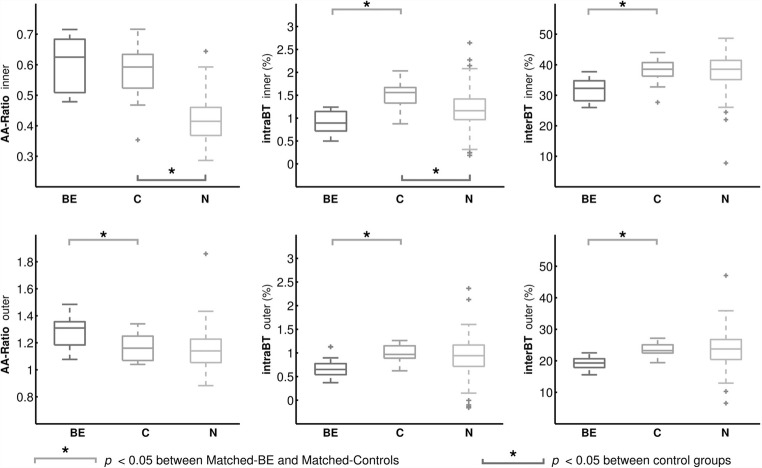
Distributions of airway–artery ratio, intra-branch tapering and inter-branch tapering for each dataset. BE, Matched-Bronchiectasis; C, Matched-Controls; N, NormalCT. Note that bronchiectatic subjects show significantly lower airway tapering than controls

Figure 3 shows the distribution of each image biomarker, with the descriptive statistics detailed in Table 2.

**Table 2:** Summary of AA ratio and tapering biomarkers and comparison between the three datasets

		Matched-Bronchiectasis	Matched-Controls	NormalCT	BE vs C	C vs N
AA ratio	Inner	0.62 [0.51, 0.68]	0.59 [0.52, 0.63]	0.41 [0.37, 0.46]	0.4705	*0.0000*
AA ratio	Outer	1.31 [1.18, 1.36]	1.16 [1.07, 1.25]	1.14 [1.05, 1.23]	*0.0226*	0.5173
Intra-branch tapering	Inner	0.89 [0.72, 1.14]	1.56 [1.33, 1.67]	1.16 [0.97, 1.42]	*0.0003*	*0.0049*
Intra-branch tapering	Outer	0.65 [0.54, 0.77]	0.97 [0.89, 1.15]	0.94 [0.72, 1.17]	*0.0029*	0.6757
Inter-branch tapering	Inner	32.28 [28.19, 34.75]	38.54 [36.26, 40.73]	38.54 [35.12, 41.43]	*0.0024*	0.9778
Inter-branch tapering	Outer	19.31 [17.86, 20.65]	23.23 [22.45, 25.08]	23.75 [20.41, 26.75]	*0.0005*	0.9087

The median and inter-quartile range are shown for the image biomarkers extracted from all airway–artery pairs. *p* values of the Mann–Whitney *U* test are shown for each image biomarker: between the Matched-Bronchiectasis (*BE*) and Matched-Controls (*C*); and between Matched-Controls and NormalCT (*N*). Significant values (*p* < 0.05) are italicised

Since tapering measurements do not require a paired artery, tapering measurements including all 22,275 airways (13,987 AA pairs and 8288 airways without a paired artery) are also reported in ESM e-Table 5. No significant differences in tapering were observed between measurements obtained from all airways versus measurements using only paired airways and arteries.

### Image biomarkers for bronchiectasis quantification, stratified by airway size

**Fig. 4:**
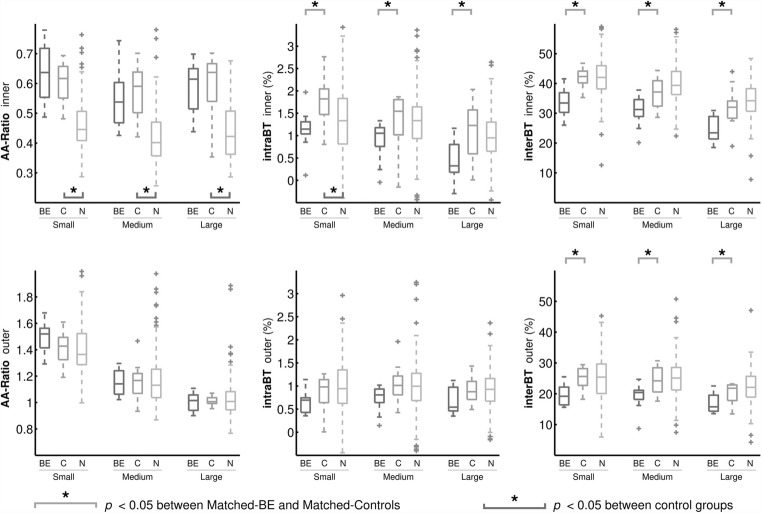
Distributions of airway–artery ratio, inter-branch tapering and intra-branch tapering for small, medium and large airways, grouped by dataset. BE, Matched-Bronchiectasis; C, Matched-Controls; N, NormalCT. Some outliers in the NormalCT that fell outside the axes range have been removed from this plot for clarity. Note that changes in airway tapering are observed across all airway sizes between bronchiectatic subjects and controls

Figure 4 shows the image biomarker distribution in the three datasets divided into airways with small, medium and large arteries (ESM e-Tables 2 to 4 for detailed values).

Inner and outer AA ratios were not significantly different between Matched-Bronchiectasis and Matched-Controls for any airway size group. In contrast, intraBT and interBT of the inner airway, and interBT of the outer airway were significantly less in the Matched-Bronchiectasis compared with those in the Matched-Controls for all airway size groups. IntraBT of the outer airway trended to be less in the Matched-Bronchiectasis group compared with that in the Matched-Controls for all airway size groups, but this finding was not significant (*p* < 0.08).

### Stability of biomarkers under varying scanning conditions

When analysing all airways, the AA ratio and intraBT of the inner airways were significantly different between the control groups with and without lung volume control (Fig. 3 and Table 2).

When airways were stratified by size, all AA ratios and intraBT for inner airways of the small artery sizes were significantly different between the spirometry-controlled subjects of the Matched-Controls and the free-breathing subjects of the NormalCT dataset (Fig. 4, and ESM e-Tables 2–4).

### Correlations with visual scoring

The median (IQRs) of the visual bronchiectasis scores were 7.45 (1.40–13.38) for the Matched-Bronchiectasis and 0.00 (0.00–0.70) for the Matched-Controls. Per-subject median values correlated well with CF-CT bronchiectasis sub-score for all biomarkers (Table 3). Only interBT and intraBT of the inner airways showed a strong and significant correlation with CF-CT bronchiectasis across all airway size groups.

**Table 3:** Correlation of the image biomarkers with visual scoring

	Inter-branch tapering			
	All	Small	Medium	Large
Inner airway	*− 0.67 (0.0004)*	*− 0.57 (0.0042)*	*− 0.49 (0.0175)*	*− 0.52 (0.0094)*
Outer airway	*− 0.65 (0.0005)*	*− 0.51 (0.0139)*	*−* 0.31 (0.1465)	*− 0.53 (0.0076)*
	Intra-branch tapering			
	All	Small	Medium	Large
Inner airway	*− 0.75 (0.0000)*	*− 0.70 (0.0002)*	*− 0.65 (0.0009)*	*− 0.60 (0.0021)*
Outer airway	*− 0.56 (0.0046)*	*−* 0.35 (0.1041)	*− 0.50 (0.0144)*	*− 0.44 (0.0334)*
	AA ratio			
	All	Small	Medium	Large
Inner airway	*0.53 (0.0083)*	*0.64 (0.0011)*	0.28 (0.1904)	0.14 (0.5142)
Outer airway	*0.75 (0.0000)*	*0.73 (0.0001)*	*0.46 (0.0272)*	0.24 (0.2631)

Spearman correlation coefficient was used between visual scores of bronchiectasis and each image biomarker was stratified by size of all the patients of the Matched-Bronchiectasis and Matched-Control datasets. Significant values (*p* < 0.05) are italicised

## Discussion

Lack of airway tapering or less tapering has long been known to be an important criterion to define bronchiectasis in clinical practice [25]. Recent developments in computer algorithms to analyse chest CTs result in more insight into tapering as a biomarker. In this study, semi-automated intra- and inter-branch airway tapering values were quantified in a cohort of normal paediatric chest CTs for control values and were compared with tapering values of children with bronchiectasis.

The first important finding of this study is that control tapering values were established on CTs of paediatric patients. No previous reported values exist for the intra-branch tapering of the inner or outer airway or the inter-branch tapering of the outer airway. Inter-branch tapering of the inner airway (also known as homothety ratio) has previously been reported in bronchial casts [3] and CT scans [26, 27] of healthy adults and ranged between 21 and 29%. These values are lower than the median inter-branch tapering of the inner airway of 38.5% reported in this study for the NormalCT dataset. A possible explanation for this difference is that the automatic diameter measurements are consistently lower than measured by human observers, leading to a higher tapering value [21]. A constant, absolute reduction in diameter along the airway tree would result in higher tapering values. Another explanation could be that the present study analysed paediatric subjects, whereas previous reporting showed tapering values for adults.

The second important finding is that tapering measures were an independent biomarker reflecting airway disease. Significantly less intra-branch and inter-branch tapering was seen in the 12 patients with bronchiectasis in comparison with that in a matched control group, for both inner and outer airway diameters. This is in concordance with the visually assessed lack of tapering in bronchiectatic airways as previously described [2, 14, 17]. In addition, tapering measurements of diseased airways correlated well with visual scores. Previous studies measuring airway tapering in bronchiectatic subjects focused solely on inter-branch tapering of the inner airway and are in concordance with this study. Odry et al found a correlation between tapering of the inner airway and visual inspection of bronchiectasis in one healthy and 8 diseased subjects [28]. Weinheimer et al found a correlation between lobar inner airway taper indices and visual scores in 144 CTs of paediatric CF patients [18].

The third important finding is that airways taper significantly less across all airway sizes in patients with bronchiectasis compared with that in the matched controls. In contrast, the AA ratio was only significantly increased in the group of airways with arteries of patients with bronchiectasis. These findings suggest that tapering has potential to add information to our current understanding of the pathophysiology of diffuse airways disease. Abnormally dilated peripheral airway might coincide with less tapering in the airway branches immediately before the dilated branch or all the way up to the central airways (Fig. 1). Our study showed less airway tapering to be not only present in the dilated branch but also noticeably less tapered in the more central airways. As such, abnormal structural changes related to bronchiectasis in the smaller airways are present in larger airways as well and can be detected using our automated analysis. Differences in airway tapering observed in medium and large airways (grouped by accompanying artery size) indicate that differences between groups are not driven by a selection bias, where abnormally dilated airways are more predominant in the airway group with small arteries of diseased subjects. From a clinical point of view, detecting structural changes in the larger airways could mean that airway disease progression can be measured with greater precision, in an earlier stage, or on CT scans with lower radiation exposure such as the CT protocols used in infants [29]. This is important for lung diseases that are affected by bronchiectasis, since bronchiectasis is an irreversible condition and when present treatment needs to be initiated to prevent further progression [30].

The final important finding of this study is that outer airway measures seem to be affected less by inspiration level during CT acquisition. Significant differences in AA ratio and intraBT of the inner airway between the spirometry-controlled inspiration scans of the Matched-Controls and the CTs acquired without lung volume control of the NormalCT dataset were observed, while none of the outer airway measurements differed significantly between control groups. This is in agreement with previous studies reporting the outer airway diameter to be more robust to variations in lung volume and scanning protocol [1].

This study has several limitations. First, the number of subjects with bronchiectasis analysed is small and consisted of 11 patients with CF and one patient with CVID. However, nearly 14,000 airway–artery pairs were analysed, providing sufficiently accurate measures to observe significant differences and creating preliminary insights into tapering indices in children. In addition, both diseases lead to recurrent infections inducing structural airway abnormalities including bronchiectasis. Secondly, the control patients might have been affected by disease. All control subjects were reported by two independent radiologists who discarded any subject with signs of lung abnormalities or bronchiectasis on CT, but we cannot exclude that subtle signs of lung abnormality may have been missed. In this case, the actual difference between bronchiectatic and control groups would have been larger than perceived in this study. Additionally, the scans of the subjects in the NormalCT datasets are more heterogeneous as the subjects have a wider spread age ranges, CT acquisitions were made during different lung inspiration levels and 27% of the NormalCT dataset were CT scans with contrast and different reconstruction kernels were used. We therefore adjusted for the different lung sizes and inspiration levels. The difference in CT acquisitions such as contrast and kernels would have likely influenced the measurements of the airways and arteries [31]. However, we believe tapering measures should have been affected minimally as it estimates the percentage reduction along the airway. Third, median values within a subject were analysed, and therefore pathological changes that affect only a minority of airways might have been missed. Fourth, some discrepancy was found in the results of this study and previous work using the same dataset. Significant differences in outer AA ratio between patients with bronchiectasis and control patients were found when airways were stratified by generation [1]. This differs from the current study, where no significant differences in outer AA ratio were found between diseased and controls when airways were stratified by size. This discrepancy might be explained because the median outer AA ratio of each airway group and the subject was used to assess differences between diseased and controls, while the previous study included all AA ratio measurements in a more complex mixed-models analysis, leading to higher statistical power.

In conclusion, we reported tapering control values showed that lack of tapering associated with bronchiectasis occurs nearly uniformly across all airway sizes, and that airway tapering changes can be readily observed in larger airways while changes in AA ratios cannot. Acquiring tapering measures offers two important advantages over acquiring AA ratios. First, tapering measurements do not require extraction and quantification of arteries, pairing airways with arteries or airway generation labelling. These steps are all likely to introduce errors in an automated system. Second, and more importantly, AA ratios assume that artery size is not affected by disease. This assumption might not always hold, e.g. patients with pulmonary hypertension, COPD [32] or smokers have shown to have a reduced diameter of the pulmonary arteries, leading to an increased AA ratio [33]. Tapering is a promising biomarker for lung diseases that involve the airways. The clinical significance of tapering should be investigated in more patients and in other lung diseases for its propitious potential.

## Electronic supplementary material

ESM 1 (DOCX 33 kb)

## References

[1] Kuo W, De Bruijne M, Petersen J (2017). Diagnosis of bronchiectasis and airway wall thickening in children with cystic fibrosis: objective airway-artery quantification. Eur Radiol.

[2] Newell J.D. (2008) Bronchiectasis. In: Boiselle PM, Lynch DA (Eds) CT of the Airways. Contemporary Medical Imaging. Humana Press

[3] Weibel ER, Gomez DM (1962). Architecture of the human lung. Use of quantitative methods establishes fundamental relations between size and number of lung structures. Science.

[4] King PT (2009). The pathophysiology of bronchiectasis. Int J Chron Obstruct Pulmon Dis.

[5] Loeve M, van Hal PTW, Robinson P (2009). The spectrum of structural abnormalities on CT scans from patients with CF with severe advanced lung disease. Thorax.

[6] Maarschalk-Ellerbroek LJ, de Jong PA, van Montfrans JM (2014). CT screening for pulmonary pathology in common variable immunodeficiency disorders and the correlation with clinical and immunological parameters. J Clin Immunol.

[7] Dodd JD, Souza CA, Müller NL (2006) Conventional high-resolution CT versus helical high-resolution MDCT in the detection of bronchiectasis. AJR Am J Roentgenol 187(2):414–420 10.2214/AJR.05.0723 16861546

[8] Ciet P, Serra G, Bertolo S et al (2016) Assessment of CF lung disease using motion corrected PROPELLER MRI: a comparison with CT. Eur Radiol 26(3):780–787 10.1007/s00330-015-3850-9 26024847

[9] Tepper LA, Caudri D, Utens EMWJ, van der Wiel EC, Quittner AL, Tiddens HA (2014) Tracking CF disease progression with CT and respiratory symptoms in a cohort of children aged 6-19 years. Pediatr Pulmonol 1189(February):1182–1189 10.1002/ppul.22991 24574038

[10] Tiddens HA, Rosenow T (2014) What did we learn from two decades of chest computed tomography in cystic fibrosis? Pediatr Radiol 44(12):1490–1495 10.1007/s00247-014-2964-6 25164327

[11] Bortoluzzi CF, Volpi S, D’Orazio C et al (2014) Bronchiectases at early chest computed tomography in children with cystic fibrosis are associated with increased risk of subsequent pulmonary exacerbations and chronic pseudomonas infection. J Cyst Fibros 13(5):564–571 10.1016/j.jcf.2014.03.006 24726420

[12] Wielpütz MO, Eichinger M, Weinheimer O (2013). Automatic airway analysis on multidetector computed tomography in cystic fibrosis: correlation with pulmonary function testing. J Thorac Imaging.

[13] Smith BM, Hoffman EA, Rabinowitz D (2014). Comparison of spatially matched airways reveals thinner airway walls in COPD. The Multi-Ethnic Study of Atherosclerosis (MESA) COPD Study and the Subpopulations and Intermediate Outcomes in COPD Study (SPIROMICS). Thorax.

[14] Brody AS, Kosorok MR, Li Z (2006). Reproducibility of a scoring system for computed tomography scanning in cystic fibrosis. J Thorac Imaging.

[15] Kapur N, Masel JP, Watson D, Masters IB, Chang AB (2011). Bronchoarterial ratio on high-resolution CT scan of the chest in children without pulmonary pathology: need to redefine bronchial dilatation. Chest.

[16] Mott LS, Graniel KG, Park J (2013). Assessment of early bronchiectasis in young children with cystic fibrosis is dependent on lung volume. Chest.

[17] Naidich DP, Webb WR, Muller NL, Vlahos I, Krinsky GA (2007) Chapter 5. Airways. In: Naidich DP, Webb WR, Muller NL, Vlahos I, Krinsky GA (Eds) Computed tomography and magnetic resonance of the thorax. Lippincott Williams & Wilkins 5:453–556

[18] Weinheimer O,WielpützMO, Konietzke P et al (2017) Fully automated lobe-based airway taper index calculation in a low dose MDCT CF study over 4 time-points. SPIE Med Imaging Image Process 101330

[19] Kuo W, Ciet P, Andrinopoulou E-R et al (2018) Reference values for central airway dimensions on CT images of children and adolescents. AJR Am J Roentgenol 210(2):423–430 10.2214/AJR.17.18597 29261353

[20] Kuo W, Andrinopoulou E-R, Perez-Rovira A, Ozturk H, de Bruijne M, Tiddens HA (2017) Objective airway artery dimensions compared to CT scoring methods assessing structural cystic fibrosis lung disease. J Cyst Fibros 16(1):116–123 10.1016/j.jcf.2016.05.015 27343002

[21] Perez-Rovira A, Kuo W, Petersen J, Tiddens HA, de Bruijne M (2016) Automatic airway–artery analysis on lung CT to quantify airway wall thickening and bronchiectasis. Med Phys 43(10):5736–5744 10.1118/1.4963214 27782697

[22] Lo P, Sporring J, Ashraf H, Pedersen JJH, de Bruijne M (2010). Vessel-guided airway tree segmentation: a voxel classification approach. Med Image Anal.

[23] Petersen J, Nielsen M, Lo P (2014). Optimal surface segmentation using flow lines to quantify airway abnormalities in chronic obstructive pulmonary disease. Med Image Anal.

[24] Kuo W, Thomas S, Rosenow T, Andrinopoulou ER, Ranganathan S, Turkovic L, et al. (2017) Quantitative assessment of airway dimensions in young children with cystic fibrosis lung disease using chest computed tomography. Pediatr Pulmonol 52(11):1414–1423 10.1002/ppul.23787 28881106

[25] Kang EY, Miller RR, Müller NL (1995). Bronchiectasis: comparison of preoperative thin-section CT and pathologic findings in resected specimens. Radiology.

[26] Tawhai MH, Hunter P, Tschirren J, Reinhardt J, McLennan G, Hoffman EA (2004) CT-Based geometry analysis and finite element models of the human and ovine bronchial tree. J Appl Physiol (1985) 97(6):2310–2321 10.1152/japplphysiol.00520.2004 15322064

[27] Montaudon M, Desbarats P, Berger P, de Dietrich G, Marthan R, Laurent F (2007). Assessment of bronchial wall thickness and lumen diameter in human adults using multi-detector computed tomography: comparison with theoretical models. J Anat.

[28] Odry BJ, Kiraly AP, Novak CL, Naidich DP, Lerallut JF (2006) Automated airway evaluation system for multi-slice computed tomography using airway lumen diameter, airway wall thickness and broncho-arterial ratio. Proc. SPIE 6143, Medical Imaging 2006: Physiology, Function, and Structure from Medical Images, 61430Q

[29] Sly PD, Brennan S, Gangell C (2009). Lung disease at diagnosis in infants with cystic fibrosis detected by newborn screening. Am J Respir Crit Care Med.

[30] Stick S, Tiddens H, Aurora P (2013). Early intervention studies in infants and preschool children with cystic fibrosis: are we ready?. Eur Respir J.

[31] Dettmer S, Entrup J, Schmidt M, de Wall C, Wacker F, Shin H (2012). Bronchial wall thickness measurement in computed tomography: effect of intravenous contrast agent and reconstruction kernel. Eur J Radiol.

[32] Chaouat A, Naeije R, Weitzenblum E (2008). Pulmonary hypertension in COPD. Eur Respir J.

[33] Diaz AA, Young TP, Maselli DJ (2017). Quantitative CT measures of bronchiectasis in smokers. Chest.

