# Supplementary material for: Airway tapering: an objective image biomarker for bronchiectasis
Source: Eur Radiol. 2020 Feb 5;30(5):2703–11. doi: 10.1007/s00330-019-06606-w (PMC7160094; doi:10.1007/s00330-019-06606-w)
Supplement: Supplementary file 1 — (DOCX 33 kb) [file 330_2019_6606_MOESM1_ESM.docx]

ELECTRONIC SUPPLEMENTARY MATERIAL

**Airway tapering: an objective image biomarker for bronchiectasis**

**MATERIALS AND METHODS**

**NormalCT:**

A subset of 156 CTs from 3 different centres were ready to be analysed at the time of this study: Barcelona (Spain), Boston (United States), and Utrecht (the Netherlands). 42 Scans (27.10%) were obtained with contrast. 82 Scans (52.90%) were not spirometry-controlled or pressure-controlled. No other information was available regarding scanning protocol.

**Automatic quantification of airways and arteries**

Initial coarse segmentations of airway lumen for both Matched datasets, and for arteries in all scans, were obtained automatically using a voxel classification approach.^3^

For the NormalCT dataset, where large variability exists between CTs in terms of scanning protocols and levels of inspiration, we opted for a semi-automated approach to obtain the initial coarse segmentation of airway lumen. First an automatic segmentation was obtained using the region growing algorithm tool of Myrian® (v1.16.2, Lung XP module) image analysis platform (Intrasense) and then the segmentation was refined manually using the same platform to include all airway branches and remove false positives. A full CT was annotated in around 1 hour.

All initial segmentations were automatically refined using an optimal surface graph-cut method to detect the inner and outer surfaces of the airways and the artery surfaces.^4^ Next, artery and inner and outer airway diameter measurements were extracted every 0.5 mm along the centrelines. Diameter measurements of airway and artery branches shorter than 5 mm were not considered reliable and were discarded. Airway and artery branches located in close proximity and with similar orientation and size were automatically paired together as airway-artery pairs, using the formulation described in Perez-Rovira *et al.*^5^

**RESULTS**

**Image biomarkers for bronchiectasis quantification, stratified by airway size**

e-Tables 3 to 5 detail the descriptive statistics of the Figure 4 shown in the main document. In these the median and distribution of each image biomarker are shown for each datasets divided in small, medium, and large airways.

**REFERENCES**

1 Kuo W, de Bruijne M, Petersen J, et al. Diagnosis of bronchiectasis and airway wall thickening in children with cystic fibrosis: Objective airway-artery quantification. European Radiology. 2017. [*Ahead of print*]

2 Brody AS, Kosorok MR, Li Z, et al. Reproducibility of a scoring system for computed tomography scanning in cystic fibrosis. *J Thorac Imaging* 2006;21(1):14–21

3 Lo P, Sporring J, Ashraf H, Pedersen J, et al. Vessel-guided airway tree segmentation: A voxel classification approach. *Med Image Anal* 2010;14(4):527–538.

4 Petersen J, Nielsen M, Lo P, et al. Optimal surface segmentation using flow lines to quantify airway abnormalities in chronic obstructive pulmonary disease. *Med Image Anal* 2014;18(3):531-541.

5 Perez-Rovira A, Kuo W, Petersen J, et al. Automatic airway-artery analysis on lung CT to quantify airway wall thickening and bronchiectasis. Medical physics 2016;43(10):5736-5744.

**e-TABLES**

***e-Table 1.*** *Scan parameters used to obtain the CTs.*

|  | **Matched-Bronciectasis** | **Matched-Controls** | **NormalCT** |
| --- | --- | --- | --- |
| **Scanner model** | SOMATOM Definition Flash | SOMATOM Definition Flash | Brilliance 16P (x18), Brilliance 64 (x90), Mx8000 IDT 16 (x1), SOMATOM Definition AS+ (x12), Sensation 16 (x2), Sensation 64 (x19) |
| **Slice thickness (mm)** | 1.00 (0.75, 1.00) | 1.00 (1.00, 1. 00) | 1.50 (0.75, 2.00) |
| **Reconstruction increment (mm)** | 0.60 (0.30, 1.00) | 0.80 (0.60, 0.80) | 1.00 (0.40, 1.50) |
| **Reconstruction kernel** | B70f (x5), B75f (x7) | B75f (x5), I70f\3 (x7) | B (x5), B20f (x5), B30f (x1), B50f (x4), B60f (x7), B70f (x16), C (x20), D (x1), L (x14), YA (x81), YB (x2) |
| **Tube voltage (kV)** | 80 (80 - 80) | 100 (80, 120) | 120 (80, 120) |
| **CTDI** | 0.74 (0.57, 0.83) | 0.76 (0.32, 1.13) | 1.90 (0.40, 17.60) * |
| *Values are expressed as: median (min, max). Subjects from the NormalCT cohort were scanned in 6 different scanners* *without lung volume control.* * *21 scans did not include CTDI data* | | | |

***e-Table 2.*** *Median, lower and upper quartiles, and p-values between groups for image biomarkers extracted from small airways paired to an artery.*

|  |  | **Matched-Bronchiectasis (BE)** | **Matched-Controls (C)** | **NormalCT (N)** | **BE vs C** | **C vs N** |
| --- | --- | --- | --- | --- | --- | --- |
| **AA-Ratio** | inner | 0.64 [0.55, 0.72] | 0.62 [0.55, 0.66] | 0.45 [0.41, 0.51] | 0.3099 | **0.0000** |
| **AA-Ratio** | outer | 1.52 [1.41, 1.56] | 1.43 [1.32, 1.49] | 1.36 [1.29, 1.52] | 0.0605 | 0.7304 |
| **intra-branch tapering** | inner | 1.14 [1.04, 1.31] | 1.82 [1.47, 2.04] | 1.33 [0.81, 1.83] | **0.0028** | **0.0414** |
| **intra-branch tapering** | outer | 0.69 [0.43, 0.75] | 0.98 [0.64, 1.14] | 0.94 [0.63, 1.35] | 0.0794 | 0.8461 |
| **inter-branch tapering** | inner | 33.42 [30.29, 36.89] | 42.32 [40.19, 44.15] | 42.02 [38.17, 45.95] | **0.0006** | 0.8968 |
| **inter-branch tapering** | outer | 19.18 [16.39, 22.16] | 25.60 [22.70, 28.16] | 25.39 [20.08, 29.65] | **0.0028** | 0.9612 |

***e-Table 3.*** *Median, lower and upper quartiles, and p-values between groups for image biomarkers extracted from medium airways paired to an artery.*

|  |  | **Matched-Bronchiectasis (BE)** | **Matched-Controls (C)** | **NormalCT (N)** | **BE vs C** | **C vs N** |
| --- | --- | --- | --- | --- | --- | --- |
| **AA-Ratio** | inner | 0.54 [0.47, 0.60] | 0.59 [0.50, 0.64] | 0.40 [0.36, 0.47] | 0.4060 | **0.0000** |
| **AA-Ratio** | outer | 1.14 [1.06, 1.24] | 1.17 [1.07, 1.22] | 1.13 [1.04, 1.25] | 0.9755 | 0.8498 |
| **intra-branch tapering** | inner | 1.05 [0.76, 1.18] | 1.54 [1.02, 1.80] | 1.33 [0.94, 1.64] | **0.0247** | 0.4786 |
| **intra-branch tapering** | outer | 0.81 [0.64, 0.93] | 1.01 [0.81, 1.22] | 1.00 [0.69, 1.28] | 0.0605 | 0.8278 |
| **inter-branch tapering** | inner | 31.26 [28.90, 34.58] | 37.13 [32.37, 40.93] | 39.35 [36.26, 44.04] | **0.0210** | 0.0849 |
| **inter-branch tapering** | outer | 20.41 [18.14, 21.12] | 24.19 [20.58, 28.42] | 25.08 [21.23, 28.52] | **0.0289** | 0.7511 |

***e-Table 4.*** *Median, lower and upper quartiles, and p-values between groups for image biomarkers extracted from large airways paired to an artery.*

|  |  | **Matched- Bronchiectasis (BE)** | **Matched-Controls (C)** | **NormalCT (N)** | **BE vs C** | **C vs N** |
| --- | --- | --- | --- | --- | --- | --- |
| **AA-Ratio** | inner | 0.61 [0.51, 0.65] | 0.64 [0.54, 0.67] | 0.42 [0.36, 0.51] | 0.5444 | **0.0000** |
| **AA-Ratio** | outer | 1.02 [0.94, 1.06] | 1.01 [0.99, 1.04] | 1.01 [0.95, 1.08] | 0.8852 | 0.9679 |
| **intra-branch tapering** | inner | 0.32 [0.18, 0.80] | 1.23 [0.59, 1.57] | 0.95 [0.65, 1.30] | **0.0086** | 0.2633 |
| **intra-branch tapering** | outer | 0.54 [0.46, 0.97] | 0.88 [0.71, 1.10] | 0.93 [0.67, 1.16] | 0.0606 | 0.9038 |
| **inter-branch tapering** | inner | 23.41 [21.38, 28.89] | 32.01 [28.31, 34.10] | 34.20 [30.67, 38.24] | **0.0086** | 0.1699 |
| **inter-branch tapering** | outer | 15.75 [14.32, 19.54] | 21.78 [17.79, 22.85] | 22.08 [18.90, 25.65] | **0.0262** | 0.1532 |

***e-Table 5.*** *Median, lower and upper quartiles, and p-values between groups of image biomarkers extracted from all airways, including airways not paired to artery.*

|  |  | **Matched-Bronchiectasis (BE)** | **Matched-Controls (C)** | **NormalCT (N)** | **BE vs C** | **C vs N** |
| --- | --- | --- | --- | --- | --- | --- |
| **intra-branch tapering** | inner | 0.91 [0.73, 1.12] | 1.55 [1.29, 1.71] | 1.10 [0.92, 1.33] | **0.0007** | **0.0006** |
| **intra-branch tapering** | outer | 0.62 [0.54, 0.78] | 1.03 [0.89, 1.14] | 0.98 [0.79, 1.18] | **0.0009** | 0.6912 |
| **inter-branch tapering** | outer | 30.62 [26.88, 32.01] | 36.73 [34.84, 38.13] | 36.76 [33.03, 39.26] | **0.0011** | 1.0000 |
| **inter-branch tapering** | outer | 18.40 [18.19, 19.29] | 23.01 [22.23, 24.82] | 22.60 [19.98, 25.28] | **0.0001** | 0.3971 |
